# Geographical Health District and Distance Traveled Influence on Clinical Status at Admission of Patients with Gestational Trophoblastic Disease

**DOI:** 10.1055/s-0043-1772179

**Published:** 2023-08-18

**Authors:** Valdete Aparecida Ribeiro da Silva, Izildinha Maestá, Roberto Antonio de Araújo Costa, Aline de Ávila Campos, Antonio Braga, Neil Horowitz, Kevin M. Elias, Ross Berkowitz

**Affiliations:** 1 Postgraduation Program in Tocogynecology, Botucatu Medical School, São Paulo State University, Botucatu, SP, Brazil.; 2 Botucatu Trophoblastic Disease Center, Botucatu Medical School Hospital, São Paulo State University, Botucatu, SP, Brazil.; 3 Scientific Initiation Program by the São Paulo Research Foundation, Botucatu Medical School, São Paulo, SP, Brazil.; 4 Rio de Janeiro Trophoblastic Disease Center, Maternity School of the Rio de Janeiro Federal University, Rio de Janeiro, RJ, Brazil.; 5 Division of Gynaecologic Oncology, Department of Obstetrics, Gynaecology and Reproductive Biology, New England Trophoblastic Disease Center, Brigham and Women's Hospital, Harvard Medical School, Boston, MA, USA.; 6 Division of Gynaecologic Oncology, Department of Obstetrics and Gynaecology, Brigham and Women's Hospital, Boston, MA, USA.

**Keywords:** gestational trophoblastic disease, geographical health district, distance traveled, clinical status, referral center, doença trofoblástica gestacional, distrito de saúde, distância viajada, estado clínico, centro de referência

## Abstract

**Objective** To assess the potential relationship of clinical status upon admission and distance traveled from geographical health district in women with gestational trophoblastic disease (GTD).

**Methods** This is a cross-sectional study including women with GTD from the 17 health districts from the São Paulo state (I–XVII), Brazil, referred to the Botucatu Trophoblastic Disease Center (specialized center, district VI), between 1990 and 2018. At admission, hydatidiform mole was assessed according to the risk score system of Berkowitz et al. Gestational trophoblastic neoplasia was evaluated using the International Federation of Gynecology and Obstetrics / World Health Organization (FIGO/WHO) staging/risk score. Data on demographics, clinical status and distance traveled were collected. Multiple regression analyses were performed.

**Results** This study included 366 women (335 hydatidiform mole, 31 gestational trophoblastic neoplasia). The clinical status at admission and distance traveled significantly differed between the specialized center district and other districts. Patients referred from health districts IX (β = 2.38 [0.87–3.88], *p* = 0.002) and XVI (β = 0.78 [0.02–1.55], *p* = 0.045) had higher hydatidiform mole scores than those from the specialized center district. Gestational trophoblastic neoplasia patients from district XVI showed a 3.32 increase in FIGO risk scores compared with those from the specialized center area (β = 3.32, 95% CI = 0.78–5.87, *p* = 0.010). Distance traveled by patients from districts IX (200km) and XVI (203.5km) was significantly longer than that traveled by patients from the specialized center district (76km).

**Conclusion** Patients from health districts outside the specialized center area had higher risk scores for both hydatidiform mole and gestational trophoblastic neoplasia at admission. Long distances (>80 km) seemed to adversely influence gestational trophoblastic disease clinical status at admission, indicating barriers to accessing specialized centers.

## Resumo

**Objetivo** Avaliar a possível relação entre estado clínico na apresentação e distância percorrida a partir do distrito de saúde em mulheres com doença trofoblástica gestacional.

**Métodos** Estudo transversal incluindo mulheres com doença trofoblástica gestacional dos 17 distritos de saúde do estado de São Paulo (I–XVII), Brasil, encaminhadas ao Centro de Doenças Trofoblásticas de Botucatu (distrito VI), entre 1990 e 2018. Na admissão, avaliaram-se mola hidatiforme pelo sistema de pontuação de risco de Berkowitz et al. e neoplasia trofoblástica gestacional pelo escore de risco/estadiamento Federação Internacional de Ginecologia e Obstetrícia / Organização Mundial da Saúde (FIGO/OMS). Coletaram-se dados demográficos, clínicos e distância percorrida e análises de regressão múltipla foram realizadas.

**Resultados** Este estudo incluiu 366 mulheres (335 mola hidatiforme, 31 neoplasia trofoblástica gestacional). O estado clínico na apresentação e distância percorrida diferiram significativamente entre o centro especializado e demais distritos. Nas pacientes encaminhadas pelos distritos IX (β = 2,38 [0,87–3,88], *p* = 0,002) e XVI (β = 0,78 [0,02–1,55], *p* = 0,045), os escores de mola hidatiforme foram maiores que no centro especializado. As pacientes com neoplasia trofoblástica gestacional do distrito XVI apresentaram escores FIGO 3,32 vezes maior que no centro especializado (β = 3,32, 95% CI = 0,78–5,87, *p* = 0,010). A distância percorrida pelas pacientes dos distritos IX (200km) e XVI (203,5km) foi significativamente maior do que a percorrida pelas pacientes do centro especializado (76km).

**Conclusão** Pacientes de distritos de saúde fora da cobertura do centro especializado apresentaram escores de risco mais alto para mola hidatiforme e para neoplasia trofoblástica gestacional na admissão. Longas distâncias (>80 km) pareceram influenciar negativamente o estado clínico da doença trofoblástica gestacional na apresentação, indicando barreiras no acesso a centros especializados.

## Introduction

Gestational trophoblastic disease (GTD) is the term given to a group of rare tumors that arise during pregnancy, which are associated with abnormal proliferation of trophoblastic cells and increased secretion of human chorionic gonadotropin (hCG). Hydatidiform mole (HM), also known as pre-malignant GTD, can be either complete (no embryonic/fetal tissue is present) or partial (some embryonic/fetal tissue develops). Gestational trophoblastic neoplasia (GTN), is the malignant form of GTD that encompasses different histopathological types (invasive mole, choriocarcinoma, placental-site trophoblastic tumor, and epithelioid trophoblastic tumor).

The clinical characteristics of GTD patients at presentation at a specialized center are considerably influenced by social and economic factors, as well as barriers to healthcare access, which lead to patient presentation at later stage disease. 1 2 3 A potential cause for a less favorable outcome may be the distance required to travel to a specialized center. 3 4 Given the rarity of GTD, centralizing care for this condition has been internationally promoted as a means to improve care. However, disadvantages such as longer travel times are not usually taken into account.

To ensure universal health coverage and equity in the country, Brazil has implemented the regionalization of health services. 5 The Botucatu Trophoblastic Disease Center (BTDC) of the São Paulo State University (UNESP), provides tertiary care primarily to residents in the state Health District-VI, which encompasses 68 municipalities and covers an area of 26.790,1 km ^2^ , with an estimated population of 1.8 million inhabitants. Nonetheless, women with GTD from other localities are also treated at BTDC, regardless of their home residence.

Regionalizing health care, however, has not guaranteed equity in the treatment of GTD in Brazil because the disease cases of a region must be treated within the patient's own region of residence, and not all regions have a center specialized in GTD. Consequently, women with GTD are often referred to centers very far from where they live. Additionally, the efficiency of the referral system relies on the first physician seeing the patient being able to identify GTD. 1 Since this is not always the case, the patient may be subjected to a pilgrimage from service to service before being referred to a specialized center. This is a very bureaucratic and time-consuming process that has economic implications to the patient who cannot always pay the costs of transportation and lodging.

Within this framework, the objective of this study was 3-fold:

to determine the residence of the GTD patients referred to BTDC;
to compare the health districts referring GTD patients to our center regarding patient demographic characteristics, clinical status at admission, and distance traveled;
to assess the potential association between clinical status at admission and distance traveled among patients with HM or GTN (gestational trophoblastic neoplasia) referred to BTDC.

## Methods

This cross-sectional study included women with GTD referred for initial treatment to the Botucatu Trophoblastic Disease Center (BTDC) from health districts in the state of São Paulo, Brazil, between 1990 and 2018. Although women from all over the country can be referred to our center, those from other Brazilian states were not included.

The BTDC is affiliated with a public state university, and as such, provides multimodality treatment, and follow-up with multidisciplinary management, as well as chemotherapy drugs, all free of charge to women with GTD. This study was approved by the Research Ethics Committee of the Botucatu Medical School, UNESP (CAAE: 96348318.5.0000.5411).

The clinical diagnosis of HM was based on ultrasound findings suggestive of complete hydatidiform mole (CHM) or partial hydatidiform mole (PHM), and pre-uterine evacuation hCG level. The clinical diagnosis of CHM was based on ultrasound images showing a heterogeneous uterine mass with cystic spaces, vesicles, or hydropic villi, 6 while PHM was diagnosed when ultrasound findings indicated the presence of a thick placenta with several anechoic cystic lesions and, in some cases, ovular membrane and fetal growth restriction, and multiple malformations inherent to triploidy. 7 After evacuation, the sonographic diagnosis was confirmed by histopathological analysis 8 9 and immunohistochemical staining with p57 (KIP2). 10

The diagnosis of GTN was established according to the International Federation of Gynecology and Obstetrics (FIGO) criteria: 11 hCG plateau ± 10% for 4 measurements over 3 consecutive weeks (days 1,7,14, and 21); hCG level rise > 10% for 3 consecutive weekly measurements over at least 2 weeks (days 1, 7, and 14); hCG elevated for ≥ 6 months after evacuation or histologic diagnosis of choriocarcinoma.

Demographics, data on clinical status at admission, and health district of the patients' residence were collected from paper-based and electronic medical records.

The distance (km) traveled to reach the BTDC was estimated using Google Maps (Google LLC., Mountain View, CA, USA), considering the documented residential address of the patient and the address of the Botucatu Medical School Hospital, where the center is located.

According to the National Council of Health Secretaries, 5 the state of São Paulo is subdivided into 17 health districts. Thus, the health district of residence of all GTD patients was identified at admission based on each patient's municipality of residence.

The clinical status of patients with HM and GTN upon admission were considered as outcome variables. The HM level was assessed according to the molar pregnancy risk score system proposed by Berkowitz et al., 12 which is based on clinical, laboratory and radiologic findings (S1). The risk score for molar pregnancy included the following parameters: ultrasound diagnosis, uterine size for gestational age, pre-evacuation hCG, longitudinal (larger) diameter of the ovary, patient age, and presence of clinical complications. Based on the score assigned to each of these parameters, the clinical status of the patient with HM at admission was quantified using a point system ranging from 0 to 15. Patients with HM were classified as low-risk HM (score < 4) or high-risk HM (score ≥ 4) for developing GTN.

The clinical status at admission of patients referred for GTN treatment was assessed using the FIGO/WHO staging classification system and risk score (S2). The FIGO staging is performed according to the anatomical distribution of the neoplasm (stages I, II, III, and IV), and the risk scoring system uses prognostic factors for resistance to single-agent chemotherapy. A value of 0, 1, 2, or 4 is given for each risk factor, resulting in scores ranging from 0 to 25 points. Depending on the score obtained, the condition of the patient with GTN was categorized into low-risk (≤ 6 points) or high-risk GTN (≥ 7 points).

The following variables were considered as potential confounders: age (years), race (white/non-white), parity (total number of viable pregnancies), education level (elementary, high school, college/university, postgraduate), marital status (partner/no partner), employment (yes/no), and prior knowledge of GTD (yes/no).

The geographical distribution of the participants according to residence (health district) and clinical status at admission were plotted on a map of the state of São Paulo subdivided into its 17 health districts (shapefile obtained through the package geobr). Using the latitude and longitude coordinates of the study participants' home addresses provided by Google Maps, each one was scored on the map of the state of São Paulo. The thematic map according to clinical status at admission was built using the ggplot2 package. Both geobr and ggplot2 packages were used through the R software (R Foundation for Statistical Computing, Vienna, Austria) version 3.4.3.

The Chi-square, Fisher exact, or Kruskall-Wallis tests were used to compare demographic data, clinical status at admission, and traveled distance among patients from health districts VI, IX, and XVI, followed by the Dunn test for multiple comparisons. Associations of clinical status at admission of patients with HM and GTN by health district (VI, IX, and XVI) were made using multiple regression models, adjusted for confounding factors. The significance level was set at *p* < 0.05. Analyses were performed using the Statistical Package Social Sciences (SPSS, IBM Corp. Armonk, NY, USA) version 21.0, and the R software 3.4.3.

## Results

**Fig. 1:**
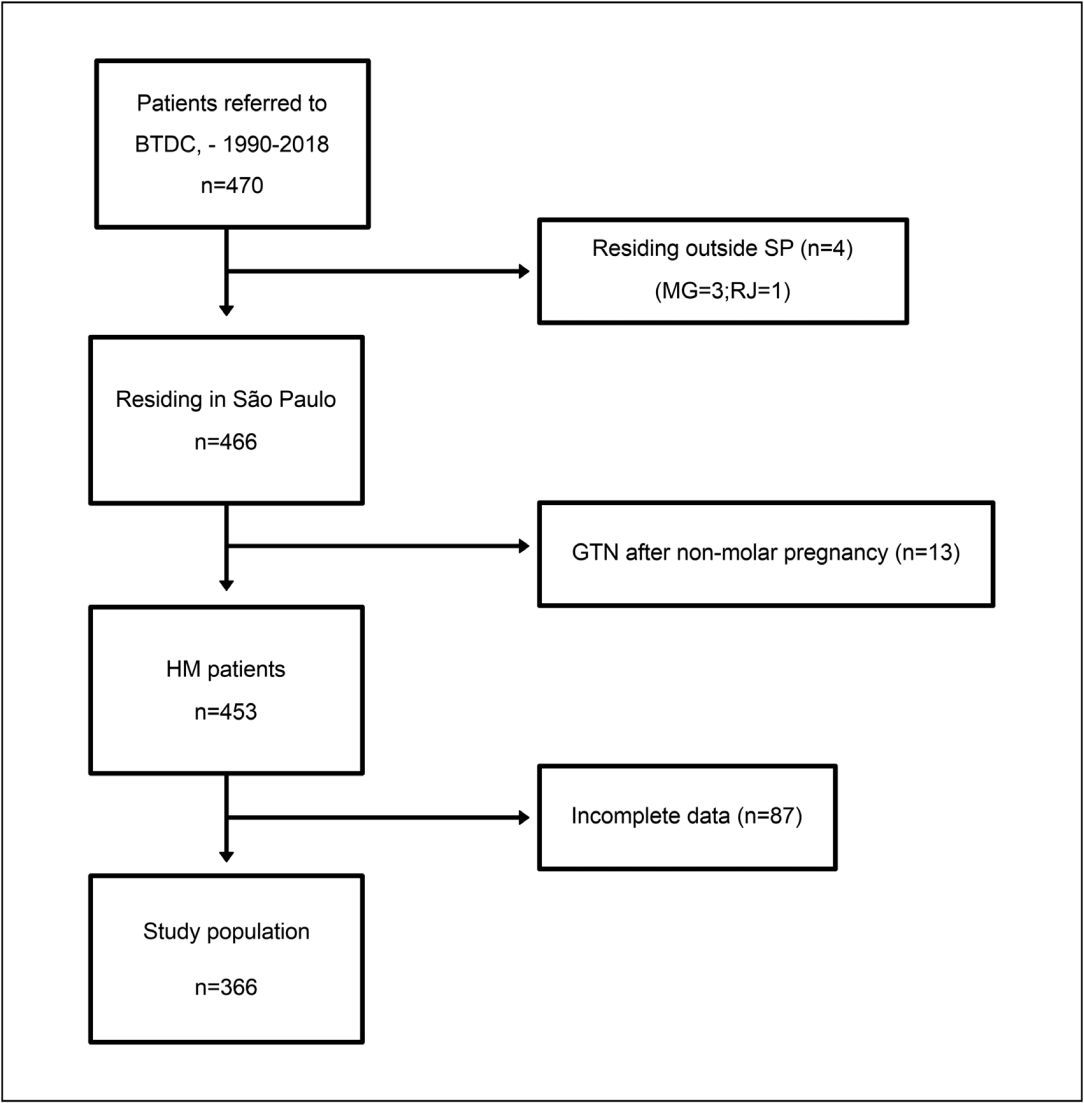
Patient flow chart.

During the study period, 470 patients were registered at BTDC. Of these, 366 met this study's inclusion criteria, and 104 were considered ineligible due to history of nonmolar pregnancy ( *n* = 13), residence outside the state of São Paulo ( *n* = 4), or missing data ( *n* = 87). Thus, the final study population consisted of 366 women: 335 with HM and 31 with GTN ( Fig. 1 ).

**Fig. 2:**
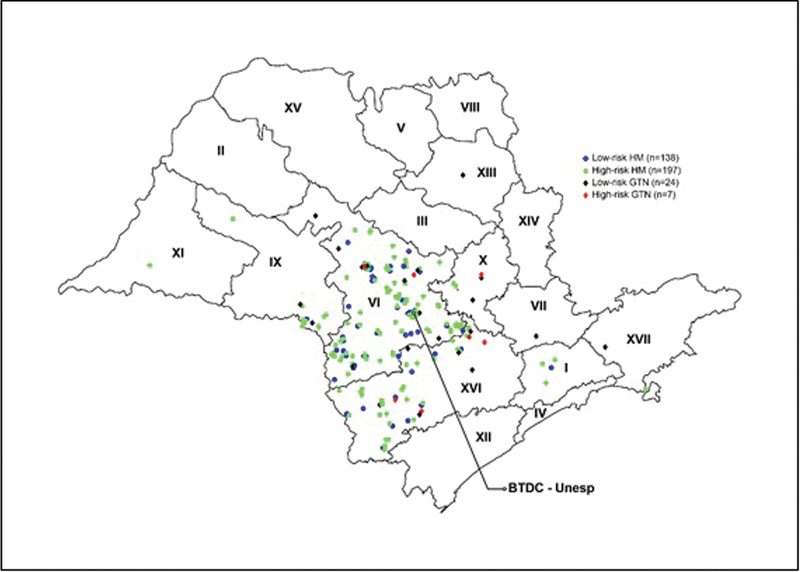
Thematic map showing the residence of the patients reffered to BTDC and their GTN status.

Of the 17 health districts in São Paulo State, 8 referred patients to BTDC. Approximately 30% of the patients referred resided in health districts outside the area covered by this center (HD VI). Furthermore, HD VI (73.5%), HD XVI (19.1%), and HD IX (4.1%) accounted for the largest number of referrals. Among GTD cases, 8.5% were referred for GTN treatment and 91.5% for molar evacuation. Notably, of the 335 patients with HM, 197 (58.8%) had the high-risk form (score ≥ 4). Among GTN patients, 22.6% were scored as high risk (≥ 7) (S3). Fig. 2 shows that the majority of the patients referred to the BTDC resided in the areas covered by health districts VI, XVI, and IX, and that the number of women with high-risk HM was greater in health district IX.

Given that only a small number of patients were from health districts I, VII, X, XII, and XIII, all the analyses presented from this point on are based on data from health districts VI, IX, and XVI, where 96% (354/366) of the study population resided.

Table 1 indicates that, overall, women from the health districts accounting for the largest number of referrals (VI, IX, and XVI) were young (median age = 23 years, min–max, 13–52), white ( *n* = 263; 74.5%), nulliparous ( *n* = 180; 50.8%), had a low education level (primary education; *n* = 179; 51.0%), and lived with a partner ( *n* = 275; 77.9%). About 30% (107) of these women were adolescents, as per the definition of the World Health Organization. 13 Among nonadolescents ( *n* = 247), less than half (49.6%) had a job. In general, prior knowledge of GTD (as assessed per the BTDC's protocol) was poor (3/354; 0.8%).

**Table 1:** Demographics, clinical status at admission and distance traveled according to health district

Variable	Overall ( *n* = 354)	HD			*p* -value
		HD VI ( *n* = 269)	HD XVI ( *n* = 70)	HD IX ( *n* = 15)	
Age (years)	23 (13–52)	22 (13–47)	25 (15–52)	19 (13–45)	0.017
10–19	107 (30.2%)	86 (32%)	12 (17.1%)	9 (60%)	
20–39	225 (63.6%)	169 (62.8%)	51 (72.9%)	5 (33.3%)	0.005
≥ 40	22 (6.2%)	14 (5.2%)	7 (10.0%)	1 (6.7%)	
Race					
White	263 (74.5%)	203 (75.7%)	48 (68.6%)	12 (80%)	0.452
Non-white	90 (25.5%)	65 (24.3%)	22 (31.4%)	3 (20%)	
Parity	0 (0–8)	0 (0–8)	0.5 (0–5)	1 (0–2)	0.551
Nulliparous	180 (50.8%)	139 (51.7%)	35 (50%)	6 (40%)	
Primiparous	103 (29.1%)	81 (30.1%)	17 (24.3%)	5 (33.3%)	0.505
Multiparous	71 (20.1%)	49 (18.2%)	18 (25.7%)	4 (26.7%)	
Education					
Elementary school	179 (51%)	144 (53.9%)	27 (39.1%)	8 (53.3%)	
Highschool	147 (41.9%)	108 (40.4%)	33 (47.8%)	6 (40%)	0.103
College / Post-graduation ^a^	25 (7.1%)	15 (5.6%)	9 (13%)	1 (6.7%)	
Had a partner	275 (77.9%)	203 (75.7%)	60 (85.7%)	12 (80.0%)	0.187
Employed (age > 19 years)	122 (49.6%)	89 (48.6%)	29 (50.9%)	4 (66.7%)	0.434
Knowledge of GTD	3 (0.8%)	2 (0.7%)	0 (0.0%)	1 (6.7%)	0.130
HM Score ( *n* = 329)	4 (0–11)	4 (0–11)	5 (0–11)	7 (2–10)	0.048
HM classification ( *n* = 329)					
Low risk	137 (41.6%)	115 (44.9%)	19 (31.7%)	3 (23.1%)	0.07
High risk	192 (58.4%)	141 (55.1%)	41 (68.3%)	10 (76.9%)	
FIGO score	3 (1–14)	3 (1–7)	4 (2–14)	1.0 (1–2)	0.085
FIGO classification ( *n* = 25)					
Low risk	19 (76%)	11 (84.6%)	6 (60%)	2 (100%)	0.373
High risk	6 (24%)	2 (15.4%)	4 (40%)	0 (0%)	
Distance traveled (km)	92 (2–325)	76.1 (2–244)	203.5 (70.9–325)	200 (159–315)	0.001
Distance traveled > 80 km	209 (59%)	125 (46.5%)	69 (98.6%)	15 (100%)	0.001

**Abbreviations:** FIGO, International Federation of Gynecology and Obstetrics; GTD, gestational trophoblastic disease; GTN, gestational trophoblastic neoplasia; HD, health district; HM, hydatidiform mole. **Notes:** Data are expressed as median (min–max) or n (%). ^a^ Only 4 patients were post-graduated; Fisher exact test, Chi-square and Kruskal-Wallis; HD XVI > HD VI > HD IX regarding age ( *p* < 0.05); HD IX > HD XVI > HD VI regarding HM risk score ( *p* < 0.05).

Regarding clinical status at admission, the median HM risk score was 4 (min–max, 0–11) and the median GTN risk score was 3 (min–max, 1–14). The median distance traveled to reach the BTDC was 92 km. More than 50% of the patients (209/354; 59%) lived at a distance > 80 km from the BTDC. No significant differences in demographic data were observed, except for age ( *p* = 0.017). Nonetheless, clinical status at admission and distance traveled significantly differed between women from the area covered by the BTDC (health district VI) and those residing outside this area. The median HM risk score was significantly higher in health district IX than in the others ( *p* = 0.048). The median distance traveled by patients from health districts IX and XVI was significantly longer compared with patients from health district VI ( *p* < 0.001). Furthermore, the percentage of long-distance travelers (> 80 km) was higher in health districts IX and XVI than in health district VI ( *p* < 0.001) ( Table 1 ).

Table 2 shows the multiple linear regression analysis of the association of HM clinical status at admission with health district (VI, IX, and XVI), adjusted for confounders. Patients referred from health districts IX (β = 2.38 [0.87–3.88], *p* = 0.002) and XVI (β = 0.78 [0.02–1.55], *p* = 0.045) had higher HM scores than those from health district VI.

**Table 2:** Adjusted multivariate analysis of the association between HM clinical status at admission and health district of residence (VI, IX and XVI)

Variable	β	95%CI		*p* -value
Health district (Ref: HD VI)				
XVI	0.78	0.02	1.55	0.045
IX	2.38	0.87	3.88	0.002
Age (years)	-0.03	-0.08	0.02	0.244
Parity	0.1	-0.21	0.41	0.516
Non-white	0.2	-0.48	0.87	0.569
Education (Ref: Elementary school)				
Secondary school	-0.65	-1.28	0.01	0.045
College/Postgraduation	-0.12	-1.43	1.19	0.86
Had a partner	-0.36	-1.09	0.33	0.326
Employed	0.2	-0.47	0.9	0.544
Knowledge of GTD	-1.68	-4.78	1.41	0.287

**Abbreviations:** CI, confidence interval; GTD, gestational trophoblastic disease; HD, health district; HM, hydatidiform mole.

Notably, the rate of high-risk HM was nonsignificantly 33% higher among women from health district VI who resided more than 80 km far from BTDC (long-distance travelers) (RR = 1.33; 95% confidence interval [CI] = 0.96–1.86, *p* = 0.088; Poisson regression) (S4). The association between long distance and high-risk HM was not calculated for health districts IX and XVI because nearly all patients from those districts lived farther than 80 km from BTDC.

Table 3 shows the multiple linear regression analysis adjusted for confounders of the association of the GTN patients' clinical status at admission with health district of residence (VI, IX, and XVI).

The GTN patients referred from health district XVI showed a 3.32 increase in FIGO risk scores when compared with those from district VI (β = 3.32, 95% CI = 0.78–5.87, *p* = 0.010). No woman with GTN had any previous knowledge about the disease, so it was not possible to estimate its effect on the risk score.

**Table 3:** Adjusted multivariate analysis of the association between GTN clinical status at admission and health district of residence (VI, IX and XVI)

Variable	β	95% CI		*p* -value
Health district (Ref: HD VI)				
XVI	3.32	0.78	5.87	0.01
IX	-1.51	-5.72	2.7	0.481
Age (years)	0.16	0	0.33	0.048
Parity	-1.56	-2.87	-0.03	0.046
Nonwhite	-0.37	-3.03	2.31	0.783
Education (Ref: Elementary school)				
Secondary school	0.83	-1.78	3.43	0.534
College/Postgraduation	-3.24	-6.97	0.5	0.089
Had a partner	-2.37	-6.5	1.76	0.26
Employed	0.16	-2.14	2.45	0.892
Knowledge of GTD	–	–	–	–

**Abbreviations:** CI, confidence interval; IFGO, International Federation of Gynecology and Obstetrics; GTN, gestational trophoblastic neoplasia. **Notes:** No GTN patient had any previous knowledge of the disease.

## Discussion

This study indicates that geographical residence influenced the clinical status of the GTD patients referred to BTDC. Considering the demographic characteristics associated with worse outcome, we observed that GTD patients referred from health districts outside the area covered by our specialized center were mostly long-distance travelers (> 80 km) and had a higher risk score compared with those residing in the BTDC (health district VI) coverage area.

Irrespectively of the health district of residence, there was no difference in demographic characteristics in the population studied. Most women were young, had no or only 1 child, had a low educational level, and were unemployed. Such unfavorable sociodemographic status has also been reported by other centers in developing countries. 2 14 15 16 17

Among the patients with HM referred to our center, 58.8% were at high-risk of molar pregnancy (score ≥ 4). Compared with women residing in the specialized center district, women from other districts had a significantly higher molar pregnancy score. This might have resulted from the fact that despite advances in the early diagnosis of HM worldwide, 18 19 20 21 ultrasound exams and hCG assays are not always readily available in developing countries. As a result, women may be diagnosed with HM by the end of the first trimester or during the second trimester of pregnancy, when many have already developed medical complications. 2 17

A substantial number of GTN patients referred to our center (22.6%) had a high-risk score (≥ 7) (high-risk GTN), particularly patients from health district XVI, which is not in the area covered by our center (VI). In the patients from health district XVI, risk scores were significantly higher (mean of 3.32 points) than in those from district VI. Notably, this 3.32 increase in the risk score is enough to change the categorization of GTN from low to high risk.

Whereas the rate of high-risk GTN among our patients was 22.6%, Maestá et al. 22 found that in South American (Brazil and Argentina) trophoblastic disease centers, 15% of the women with GTN had the high-risk form of the disease. Both of these rates are high compared with those reported by trophoblastic centers in developed countries (2.7–6.3%). 23 24

Regarding residence, approximately ⅓ of the patients referred to the BTDC were from health districts outside the area covered by our center. The visualization of the thematic map shows that districts IX and XVI were the ones who most referred GTD patients, and both border district VI, where BTDC is located. The greater influx of patients from these districts could be easily explained as the result of a shorter distance to be traveled by the patients. However, these districts are quite large, and the median distance covered by patients to obtain care at the specialized center is 200 km, which makes them long-distance travelers. Virtually all patients residing outside the coverage area of the specialized center and about half (HD VI: 46.5%) of those residing in the coverage area of the specialized center were long-distance travelers (> 80km).

Several studies have shown that long distance travel constitutes a barrier to treatment among cancer patients. 25 26 27 Travel time to health care services has also been shown to influence access, utilization and outcomes among patients with various malignancies. 28 29 30 In women with GTN, a single study evaluating the effect of distance traveled showed that long-distance travelers were significantly more likely to present with high-risk disease (relative risk [RR] = 2.4; 95% CI = 1.1–5.2). 3 Feltmate et al. 4 also noted that a distance greater than 20 miles from the patient's home to the trophoblastic disease center was associated with failure to complete hCG follow-up ( *p* = 0.001). Furthermore, the impact of distance traveled is stronger for patients of lower socioeconomic status. 25 26

Among South American women with GTN, long distances, social and economic disparities, inefficiency of the referral system, and limited GTD training may lead to late referral to a specialized center. 22 Very often, patients are not referred to an hCG follow-up program upon hospital discharge after molar evacuation, and when they are referred to the primary healthcare system, the results of postmolar serum hCG level are very frequently delayed, and misinterpretation of hCG regression curves may delay referral to a specialized center. Furthermore, patients living far away from a specialized center may cause them to postpone seeking care because of transportation issues and reluctance to miss days of work. 3

It is worth noting that the high growth fraction of the placental trophoblast causes GTD to rapidly develop. Therefore, the timely management of patients with GTD in a specialized center can reduce morbidity and mortality. Moreover, the inefficiency of the referral system, as well as the gaps in GTD training, can lead to late referral to a specialized center. However, Brazilian regulations still do not consider it an urgent/emergency condition, which leads to delays in referral. Efforts should be made to change these referral/counter-referral regulations, as well as to allow the remote management of these patients through consultation with specialists when referral to a specialized center is not possible.

The limitations of this study were those inherent to its retrospective design and possible referral bias. As our study was conducted in a tertiary center for the treatment and follow-up of women with GTD, the data collected may overrepresent the incidence of high-risk HM and GTN at presentation. Additionally, the small number of patients from each health district might have limited statistical analysis of the effects of demographic characteristics and distance traveled on clinical status at admission.

In summary, this study showed that 1) a considerable proportion of patients with GTD (∼ 30%) referred to BTDC came from health districts outside the center's geographical area of coverage; 2) women attending this center were characterized by low socioeconomic and education level, as well as unemployment status regardless of region of residence; 3) patients from health districts outside the area covered by the specialized center had higher risk scores for both HM and GTN at admission; 4) long distances (> 80 km) seemed to adversely influence the clinical status of GTD patients at admission, indicating barriers to accessing specialized centers.

## Conclusion

Patients from health districts outside the specialized center area had higher risk scores for both HM and GTN at admission. Long distances (>80 km) seemed to adversely influence GTD clinical status at admission, indicating barriers to accessing specialized centers. Further studies are warranted to determine the potential impact of geographic location and travel distance on obtaining care in a specialized center among women with GTD.
